# Hydrolysis rate constants and activation parameters for phosphate- and phosphonate-bridged phthalonitrile monomers under acid, neutral and alkali conditions

**DOI:** 10.1016/j.dib.2017.05.015

**Published:** 2017-05-10

**Authors:** Kirill S. Belsky, Artem V. Sulimov, Boris A. Bulgakov, Alexandr V. Babkin, Alexey V. Kepman

**Affiliations:** a Lomonosov Moscow State University, Department of Chemistry, Leninskie Gory, 1-3, Moscow 119991, Russia; b Lomonosov Moscow State University, Faculty of Materials Science, Leninskie Gory, 1-73, Moscow 119991, Russia; c Institute of New Carbon Materials and Technologies (INCMaT), Leninskie Gory, 1-11, Moscow 119991, Russia

**Keywords:** Hydrolysis, Phthalonitrile, Phosphoric ester, Phosphonic ester, Rate constant, Activation energy

## Abstract

Hydrolysis data for Bis(3-(3,4-dicyanophenoxy)phenyl) phenyl phosphate and Bis(3-(3,4-dicyanophenoxy)phenyl) phenylphosphonate under pH 4, 7 and 10 are presented. Conversion/time plots collected by HPLC analysis, typical chromatograms and NMR spectra of the reactions products are given. Pseudo-first order rate constants are determined for both substrates at 25, 50 and 80 °C. Activation parameters were calculated from Arrhenius equation.

**Specifications Table**

**Table:** 

Subject area	*Chemistry*
More specific subject area	*Phthalonitrile resins*
Type of data	*Tables, graph, figure*
How data was acquired	Agilent 1260 chromatographer (column ZORBAX Eclipse Plus C18); *Bruker Avance 600 at 162 MHz for*^31^*P NMR with DMSO-d6 as solvent*
Data format	*Analyzed*
Experimental factors	*Samples were diluted in acetonitrile/buffer solution -50/50 (vol) in concentration 1 mg/ml, heated on thermostat for determined time periods and analyzed with HPLC.*
Experimental features	*Withdrawn aliquots were frozen with liquid nitrogen and unfrozen prior to analysis.*
Data source location	*Moscow, Russian Federation*
Data accessibility	*Data is with this article*
Related research article	*B. A. Bulgakov, A. V. Babkin, P. B. Dzhevakov, A. A. Bogolyubov, A. V. Sulimov, A. V. Kepman, Yu G. Kolyagin, D. V. Guseva, V. Yu Rudyak and A. V. Chertovich. Low-melting phthalonitrile thermosetting monomers with siloxane- and phosphate bridges. European Polymer Journal, (84):205–217, 2016. DOI:*10.1016/j.eurpolymj.2016.09.013

**Value of the data**

• *Hydrolysis of aryl-phosphoric and aryl-phosphonic triesters is not widely studied so new kinetic data enlarge a knowledge on this subject*
• *Only one-step hydrolysis was detected under all considered pH values indicating only phthalonitrile-containing phenol elimination even in the case of phenyl phosphate substrate*
• *The data is useful for estimation of application possibilities for phthalonitrile phosphoric and phosphonic triesters as monomers for heat-resistant thermosets.*

## 1 Data

Phthalonitrile phosphoric (**1**) and phosphonic (**2**) triesters were recently introduced as very prospective monomers for thermosetting matrices for carbon fiber reinforced plastics (CFRP) manufacturing due to untypically low melt viscosities (for phthalonitriles) and increased thermo-oxidative stability [1]. Phthalonitrile thermosets are known as the most heat resistant polymers possessing heat deflection temperatures >400 °C and stable up to 520 °C [1], [2], [3], [4], [5]. Development of low-melting phthalonitrile resins suitable cost-effective technologies for CFRP manufacturing could widely extend applications of composite materials in aerospace (for production of complex-shaped parts, e.g. jet engine blades) and other applications such as high-temperature composite tooling. However, due to the presence of organic esters of phosphonic or phosphoric acids in monomer structure a question of hydrolysis resistance of **1** and **2** naturally came up. Hydrolysis of triesters of phosphoric and phosphonic acids is barely reported in literature [6], [7]. Here the data on hydrolysis kinetics of **1** and **2** in three different pH values 4, 7 and 10 is presented including rate constants and activation parameters calculated from Arrhenius equation.

Tables Table 1 and Table 2 contain experimental data describing hydrolysis of phthalonitriles **1** and **2**.

**Table 1:** Rate constants and 48 h conversion for acidic and neutral hydrolysis of 1 and 2.

**рН**	**T**	**1**		**2**	
		**k, s**^−^^**1**^	**Conversion,**^a^**%**	**k, s**^**−1**^	**Conversion**^**⁎**^**, %**
4	25	–	0.18	–	0.21
	50	–	0.22	–	0.25
	80	–	0.24	–	0.23
7	25	4.14×10^-7^	4.86	4.16×10^-7^	2.83
	50	4.05×10^-6^	49.55	4.23×10^-6^	40.78
	80	3.04×10^-5^	100 (24 h)	7.18×10^-5^	99.86
10	25	2.41×10^-4^	100 (24 h)	2.24×10^-4^	100 (24 h)
	50	7.21×10^-4^	100 (3 h)	1.71×10^-3^	100 (1 h)
	80	3.21×10^-3^	100 (1 h)	–	100 (5 min)

a Substrate (**1** or **2**) conversion after 48 h.

**Table 2:** Activation parameters for 1 and 2 hydrolysis.

**рН**	**1**		**2**	
	**Е**_**А**_**, kcal/mole**	**А, s**^**−1**^	**Е**_**А**_**, kcal/mole**	**А, s**^**−1**^
7	16.41	4.54×10^5^	19.66	5.99×10^7^
10	10.54	1.03×10^4^	–	–

## 2 Experimental design, materials and methods

### 2.1 Materials

Monomers **1** and **2** [1] and resorcinol derivative **3** [8] were obtained according to the literature methods. Phenol was obtained from Sigma Aldrich and purified by vacuum sublimation. Acetonitrile (HPLC grade) was obtained from Sigma Aldrich and used as received. Buffer solutions with pH 4, 7 and 10 were purchased from Panreac Applichem.

### 2.2 Methods

HPLC analysis was performed on Agilent 1260 chromatographer (column ZORBAX Eclipse Plus C18; Т_column_ = 30 °С; flow rate – 0.8 ml/min). Elution program is presented in Table 3. The obtained chromatograms were developed with Agilent ChemStation software.

**Table 3:** Elution program applied for LC analysis.

Time, min	Acetonitrile, %	1% HCOOH, %
0	40	60
20	85	15
30	95	5
35	95	5
45	40	60
50	40	60

### 2.3 Sample preparation and hydrolysis study

Monomers **1** and **2** were diluted in dry acetonitrile to concentration 2 mg/ml. The initial solution (10 ml) of monomer in acetonitrile was heated to a desired temperature (25, 50 or 80 °С) and poured to equal volume of preheated buffer solution (10 ml) under stirring with magnetic bar. After that flask with the reaction mixture was heated on water bath and 1 ml aliquots were withdrawn with a pipette in certain time periods, placed to 1.5 ml glass vial, sealed with septum head and placed into liquid nitrogen to stop all the chemical processes. Samples were unfrozen immediately before HPLC analysis.

In the case of hydrolysis study under pH 10 withdrawn samples were poured to 10 μl of concentrated HCl and shaken to prevent further hydrolysis and then treated as described above.

### 2.4 HPLC-analysis

Retention times for all the pure compounds were determined before measurements (Fig. 2). Series of solutions of **1**, **2** and **3** with concentrations 1.0, 0.05, 0.01, 0.005 and 0.001 mg/ml were prepared for each monomer by consequent dilution of initial solutions with acetonitrile for LC calibration. Concentrations of the investigated compounds were determined based on calibration by automatic analysis with Agilent ChemStation software.

**Fig. 1:**
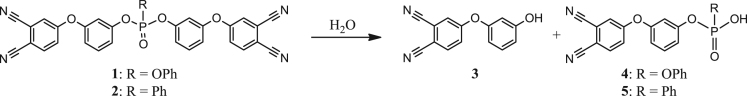
Established hydrolysis reaction of 1 and 2 under pH = 4, 7 and 10.

**Fig. 2:**
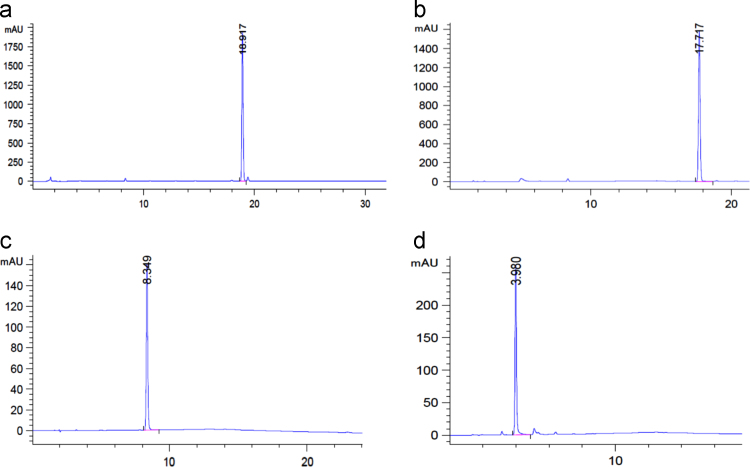
Chromatograms for: a) monomer 1; b) monomer 2; c) resorcinol derivative 3; d) phenol.

**Fig. 3:**
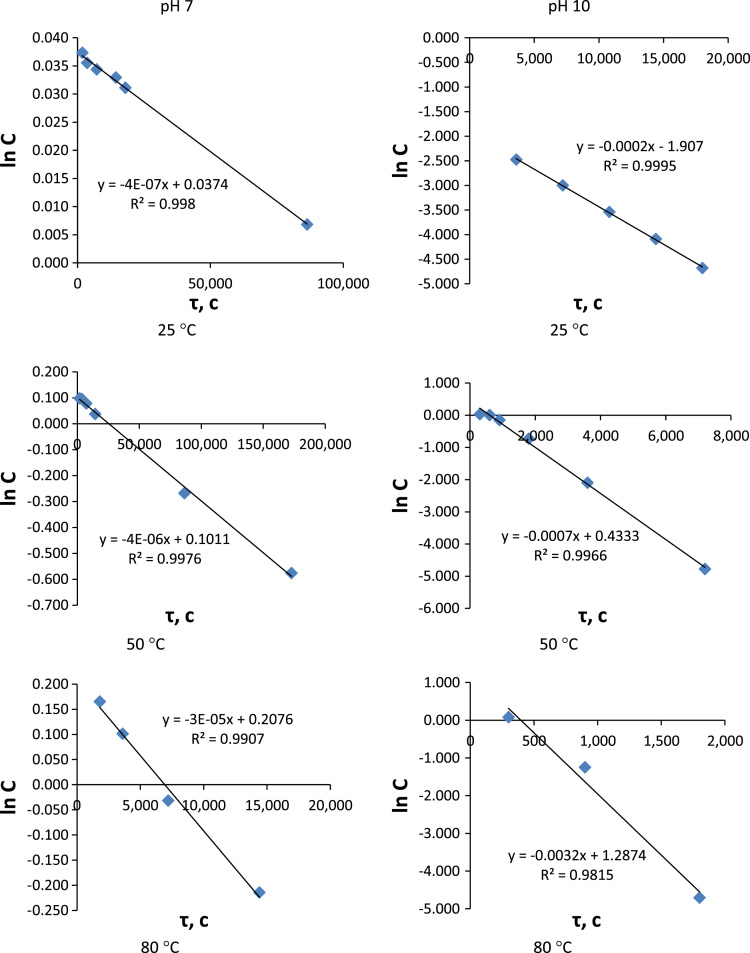
*lnC*-time plots for phthalonitrile 1 hydrolysis at different temperatures.

**Fig. 4:**
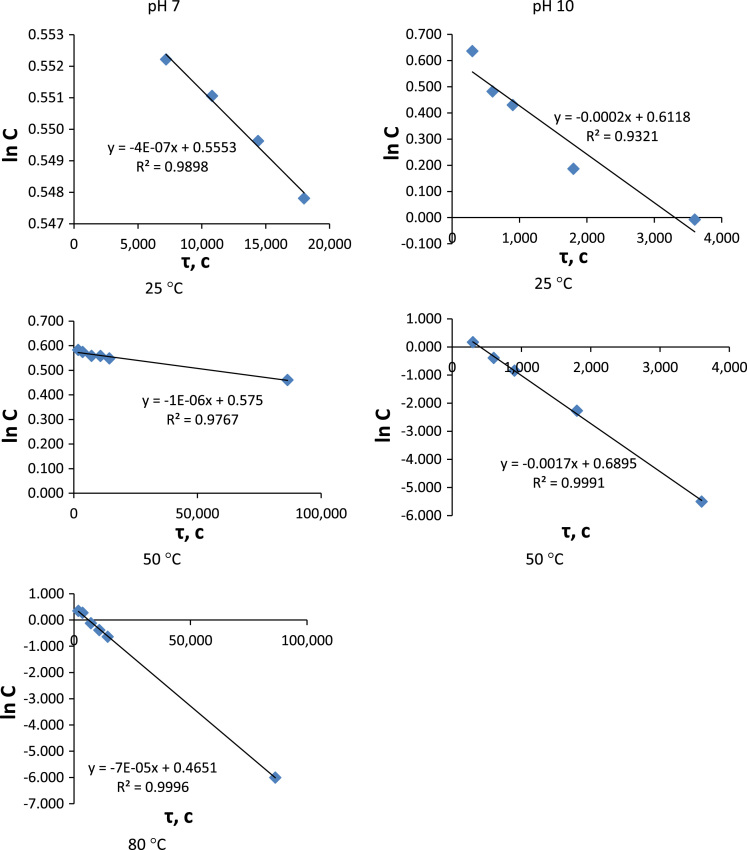
*lnC*-time plots for phthalonitrile 2 hydrolysis at different temperatures.

Several assumptions were made for the results interpretation. First of all hydrolysis of monomers **1** and **2** was considered as a pseudo-first-order reaction because water concentration in experimental conditions was sufficiently higher than substrates concentrations. It was suggested and then proven that hydrolysis passed only by the first stage (Figs. 3 and 4).

**Fig. 5:**
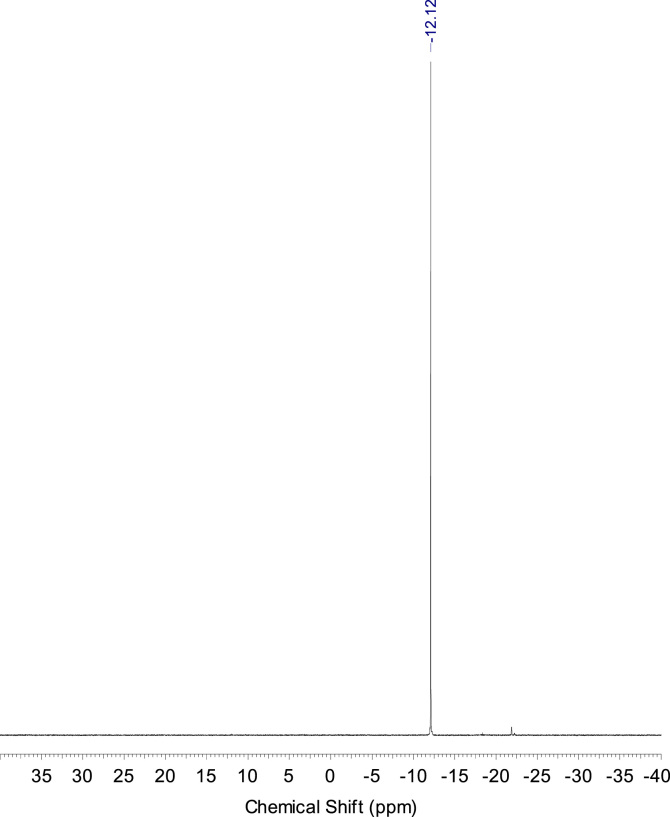
^31^P spectra of phthalonitrile **1** hydrolysis product after 48 h at 80 °C under pH 7.

**Fig. 6:**
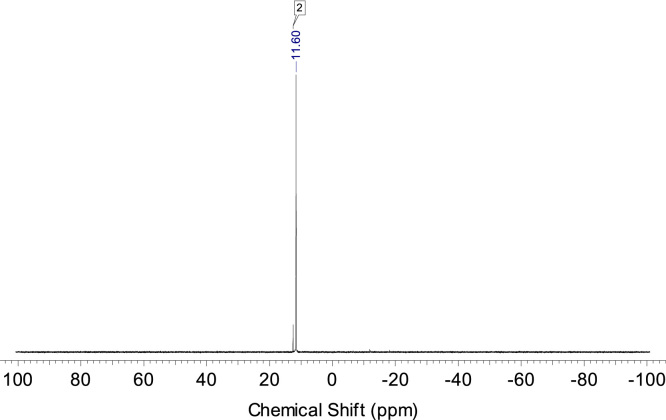
^31^P spectra of phthalonitrile 2 hydrolysis product after 48 h at 80 °C under pH 7.

The 48 h samples were acidified with HCl and then solvents were removed from samples under reduced pressure and solid residues were dissolved in DMSO-*d6* to provide NMR-study. In ^31^P NMR spectra in both cases new single signals were observed indicating formation of only one phosphorus-containing compound in each case which is in accordance with the suggested reaction path (Fig. 1). In the case of **1** product with chemical shift –12.12 ppm was detected while phosphorus resonance in the initial phthalonitrile was at –18.54 ppm [1] indicating formation of the disubstituted arylphosphate. For the phthalonitrile **2** only resonance at 11.60 ppm was observed besides the residual peak of initial monomer **2** at 12.54 ppm [1] (Fig. 5, Fig. 6).
